# SWI brush sign of cerebral parenchymal veins in central nervous system diseases

**DOI:** 10.1007/s11604-024-01723-z

**Published:** 2024-12-28

**Authors:** Seiya kishi, Masayuki Maeda, Ryota Kogue, Fumine Tanaka, Maki Umino, Naoki Toma, Hajime Sakuma

**Affiliations:** 1 Department of Radiology, Mie University Graduate School of Medicine, 2-174 Edobashi, Tsu, Mie 514-8507 Japan; 2 Department of Neuroradiology, Mie University Graduate School of Medicine, 2-174 Edobashi, Tsu, Mie 514-8507 Japan; 3 Department of Neurosurgery, Mie University Graduate School of Medicine, 2-174 Edobashi, Tsu, MIe 514-8507 Japan

**Keywords:** Brush sign, MR imaging, Susceptibility-weighted imaging, Central nervous system

## Abstract

Brush sign (BS) was first reported as prominent hypointensity of deep medullary veins and subependymal veins on T2*-weighted images at 3 T MRI in patients with acute stroke in the territory of the middle cerebral artery. Subsequently, BS in central nervous system (CNS) diseases such as moyamoya disease, cerebral venous thrombosis, and Sturge–Weber syndrome was also described on susceptibility-weighted imaging (SWI), and the clinical implications of BS were discussed. The purpose of this review is to demonstrate BS on SWI in various CNS diseases and its mechanisms in the above-mentioned diseases. We also explain the clinical implications of this finding in each disease.

## Introduction

Brush sign (BS) was first reported on T2*-weighted images at 3 T MRI in patients with acute stroke in the territory of the middle cerebral artery, defined as visualization of prominent cerebral parenchymal veins such as deep medullary veins (DM) and subependymal veins (SE) in the affected white matter [1]. This finding is speculated to be due to a blood oxygenation level-dependent (BOLD) effect caused by an increase in deoxyhemoglobin concentration in venous blood due to increased oxygen extraction in the ischemic region [2]. Subsequently, using susceptibility-weighted imaging (SWI), BS has also been reported in other central nervous system (CNS) diseases such as moyamoya disease (MMD) [3], cerebral venous thrombosis (CVT) [4], and Sturge–Weber syndrome (SWS) [5]. In this article, we demonstrate the SWI BS and its mechanisms in the above-mentioned diseases and to explain the clinical implications of this finding in each disease.

## Venous anatomy

**Fig. 1:**
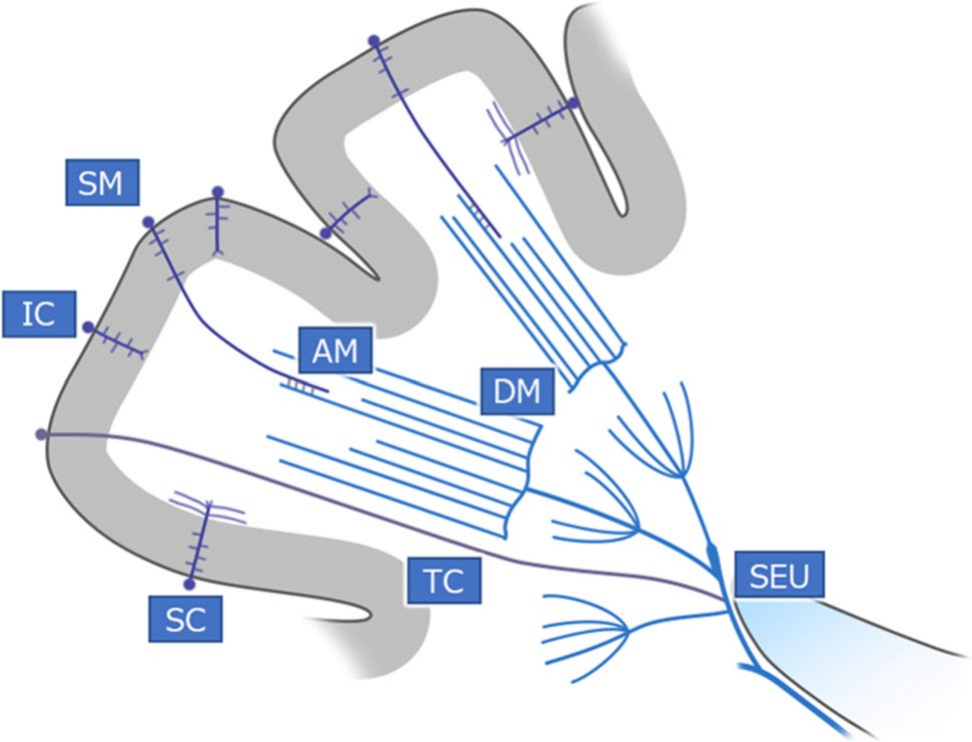
This schematic diagram shows the parenchymal venous structure of the cortex and white matter. Parenchymal venous abbreviations are: *AM* Anastomotic medullary veins, *DM* Deep medullary veins, *IC* Intracortical veins, *SEU* Subependymal union of subependymal veins, *SC* Subcortical veins, *SM* Superficial medullary veins, *TC* Transcerebral veins

Parenchymal veins in the cerebral hemispheres are divided into veins that flow on the surface (superficial venous system) and veins that flow deep (deep venous system). The former includes intracortical veins, subcortical veins, and superficial medullary veins. However, the DM extend deep and form four venous confluence zones in the fronto-parietal areas on the way to reach the SE. The superficial and deep venous systems are connected in part by anastomotic medullary veins. Some veins extend deep into the cortex and are called transcerebral veins, and the superficial and deep venous systems are connected by the anastomotic medullary veins and transcerebral veins [6]. Thus, parenchymal veins consist of intracortical veins, subcortical veins, superficial medullary veins as superficial parenchymal veins, DM and SE as deep parenchymal veins, and transcerebral and anastomotic medullary veins [6]. A schematic diagram showing the parenchymal venous structure of the cortex and white matter of the cerebral hemispheres is shown in Fig. 1.

## Susceptibility-weighted imaging (SWI)

SWI is a combination of specific sequences and processing designs developed to enhance the contrast of T2*-weighted images [7]. SWI combines a high-spatial-resolution, ideally fully flow-compensated to avoid vascular dephasing caused by flow effects and 3D gradient-echo sequence with a phase mask to highlight paramagnetic and/or diamagnetic substances [8]. SWI can detect changes in blood oxygen saturation in blood vessels with high sensitivity and track bleeding and iron deposition, and by increasing the contrast of intravenous deoxyhemoglobin, it can also improve the depiction of parenchymal veins while maintaining high resolution [9].

The effect of magnetic susceptibility can be further enhanced by fusing the intensity and phase images [10]. In the phase masking process, changing the size of the Hamming window changes the SWI contrast, so appropriate phase masking is required depending on the purpose. Images produced in this manner are reconstructed using the minimum intensity projection (minIP) technique and used in clinical practice.

## Brush sign and its synonyms in ischemic stroke

**Fig. 2:**
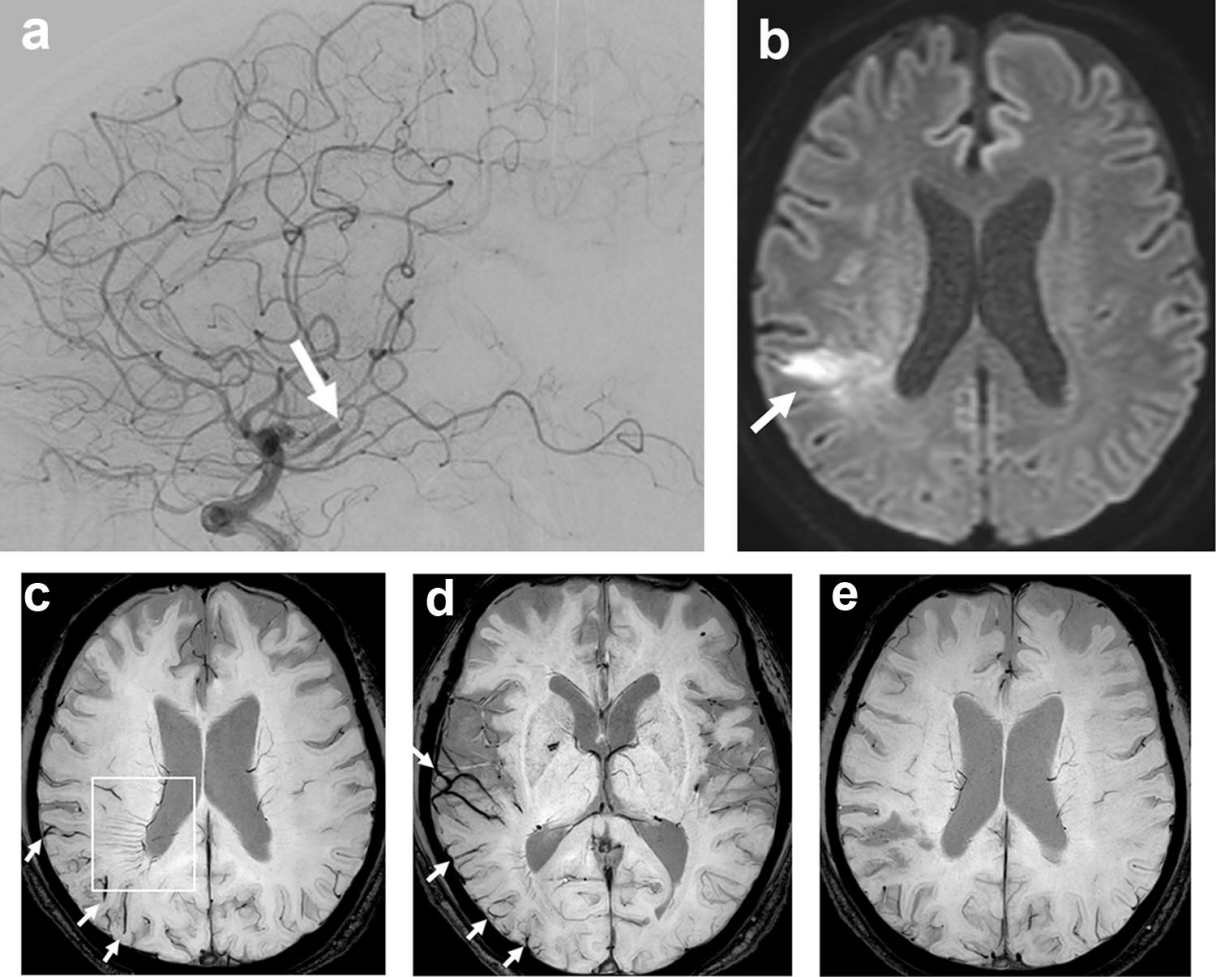
A 72-year-old man presented with left hemiparesis 7 h after onset. Angiography shows inferior branch occlusion of the right middle cerebral artery (**a**, arrow). Mechanical thrombectomy was performed but was unsuccessful. Diffusion-weighted imaging shows acute infarct in the right parietal lobe (**b**, arrow). SWI shows brush sign in the right parietal lobe (**c**, square). SWI also shows prominent cortical veins in the right temporal lobe (**d**, arrows). Seven months later, the SWI brush sign had disappeared (**e**)

BS in acute stroke is defined as prominent DM and SE [1], evidenced in areas confined to the frontoparietal regions around the lateral ventricles (Fig. 2). Besides BS, many synonyms have been proposed to explain the SWI findings of prominent hypointense veins in affected areas in cases of ischemic stroke. Several researchers have termed this as prominent vessel sign (PVS), prominent hypointense vessel sign, cortical vessel sign, ipsilateral prominent thalamostriate vein, prominent veins, asymmetrical cortical vein (CV) sign, asymmetrical medullary vein sign, prominent medullary veins, asymmetrically prominent CV, asymmetrically hypointense veins, and multiple hypointense vessels [11–23]. This increase in terminology may be due to the fact that in acute infarct, both parenchymal veins and CV are evaluated simultaneously. Some of these terms are synonymous with BS, while others refer to the different vessels involved. The PVS includes CV, DM, SE, or may even include smaller veins that contain deoxyhemoglobin [11].

**Fig. 3:**
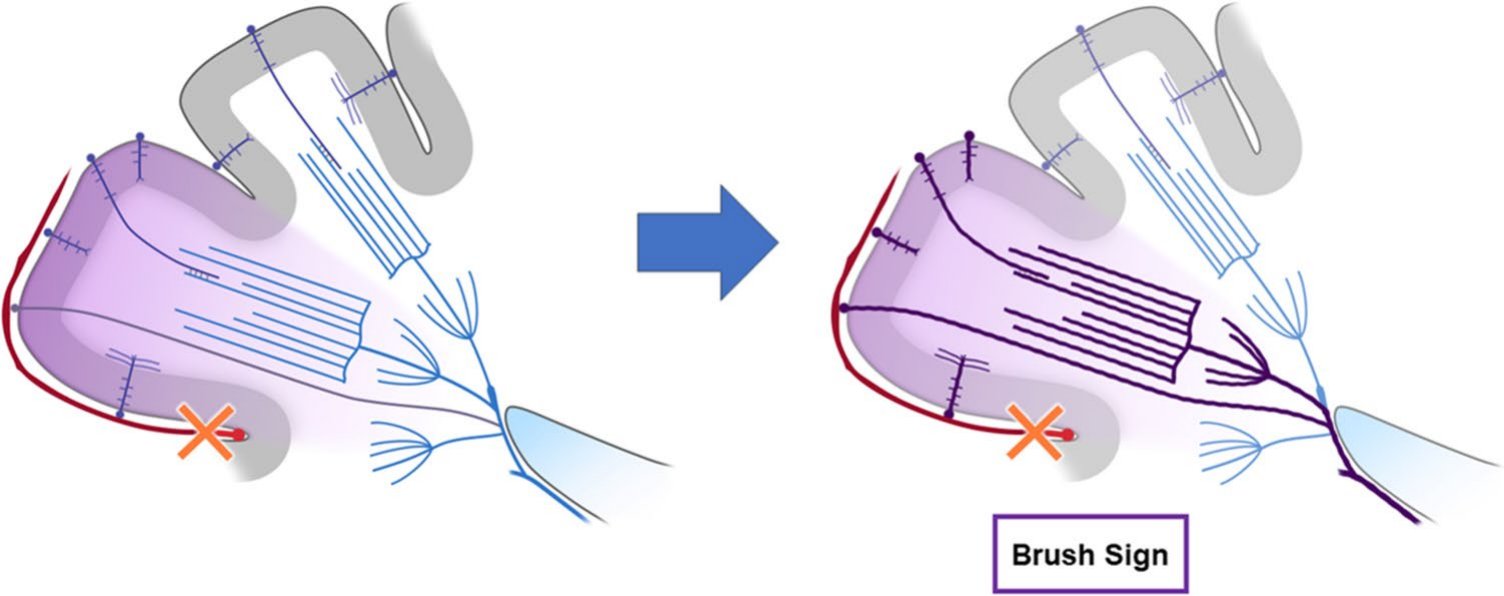
This schematic diagram shows the mechanism of the SWI brush sign in ischemic stroke. A reduction in arterial blood supply (x) increases intravenous deoxyhemoglobin in the ischemic area (area and veins highlighted in purple), resulting in the SWI brush sign, reflecting increased oxygen extraction

Prominent CV can be seen in larger regions than BS in patients with acute ischemic stroke (Fig. 2) [11, 20]. Thus, PVS can be divided into prominent CV, BS, and both according to their location. A meta-analysis showed that the presence of PVS ranged from 34 to 100% in all patients with ischemic stroke [21]. Some study described anterior circulation infarct, large vessel occlusion, and cardioembolism are independently associated with the presence of PVS on SWI [11]. In ischemic stroke, BS can be explained by a BOLD effect due to an increase in oxygen extraction rate in the acutely ischemic area (Fig. 3).

## Clinical implications of the brush sign in ischemic stroke

BS has been reported to indicate severe stroke and poor prognosis, and the fact that BS was found to be independently associated with poor prognosis after adjusting for baseline National Institutes of Health Stroke Scale (NIHSS) and other influencing factors in ordinal regression suggests that BS status has additional predictive value for early NIHSS [13]. Therefore, SWI BS is a rapid and easily assessed parameter for predicting stroke severity and can be used as an additional imaging parameter in patients with acute middle cerebral artery stroke.

Some researchers have studied the correlation between BS and hemorrhagic transformation after intravenous tissue plasminogen activator (tPA) treatment [24]. Hemorrhagic transformation on MRI was observed more frequently in the BS-positive group than in the BS-negative group. They speculated that patients who had BS even in the hyperacute phase were likely to have more severe ischemia and more vulnerable tissue than patients without BS, and more active oxygen may exist in the area with BS [24]. Therefore, this finding may predict the development of hemorrhagic transformation in stroke patients treated with tPA.

Oxygen extraction ratio is defined as the percentage of blood oxygen that tissues take up from the blood flow to maintain their functional and morphological integrity, and reflects the efficiency of oxygen utilization by tissues [25]. Increased oxygen extraction ratio indicates high oxygen extraction, and the concentration of intravenous deoxyhemoglobin increases, resulting in a positive BS. One study used BS as a surrogate for high oxygen extraction in patients undergoing mechanical thrombectomy [26]. This study showed that high oxygen extraction in patients with good collateral circulation was associated with smaller core infarcts at presentation. In contrast, patients with poor collateral circulation and high oxygen extraction may experience the greatest brain tissue damage associated with oxidative stress and, therefore, are at highest risk for core infarcts expansion. BS in patients with poor collateral circulation correlated with larger core infarct volume, suggesting that even the maximal physiological response to ischemia with high levels of oxygen extraction cannot prevent the expansion of the core infarct. Thus, the ability of collateral circulation to deliver blood to ischemic tissue may be more important for brain tissue preservation than effective oxygen extraction. This finding is consistent with previous mechanical thrombectomy trials, in which collateral circulation was found to be a significant predictor of core infarct volume and outcome after mechanical thrombectomy [27–29].

## Mechanism and clinical implications of brush sign in moyamoya disease (MMD)

MMD is an underlying intrinsic pathological disease process whose pathogenetic mechanisms are not fully understood. Within the circle of Willis, progressive stenosis or occlusion of the upper intracranial internal carotid arteries and its proximal branches occurs. This occlusion causes a compensatory vascular response, which is the formation of excess, fuzzy collateral vessels, called moyamoya vessels, at the base of the brain [30]. Based on national registry in Japan [31], pediatric and adult cases account for 28.8% and 71.2% of cases, respectively. The most common clinical presentation is infarct (29.4%), followed by transient ischemic attach (TIA) (29.1%) and hemorrhage (25.3%). Ischemic events are the most common in children (78.7%), while hemorrhage is the most common in adults (32.4%).

**Fig. 4:**
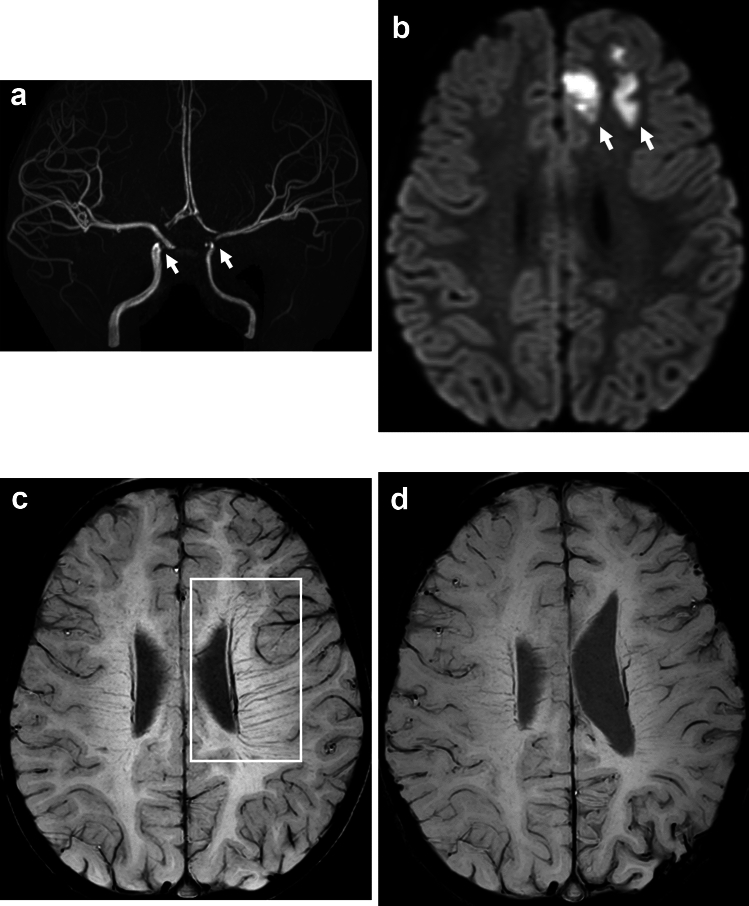
A 2-year-old boy was admitted to our hospital with right-sided hemiparesis after crying. MR angiography shows bilateral internal carotid artery stenosis 2 days after onset (**a**, arrows). Diffusion-weighted imaging shows acute infarct in the left frontal lobe (**b**, arrows). SWI shows brush sign on the left side (**c**, square). The patient was subsequently diagnosed with moyamoya disease based on angiographic findings (not shown) and underwent left-sided bypass surgery. SWI shows disappearance of the brush sign on a follow-up MRI 4 years after onset (**d**)

**Fig. 5:**
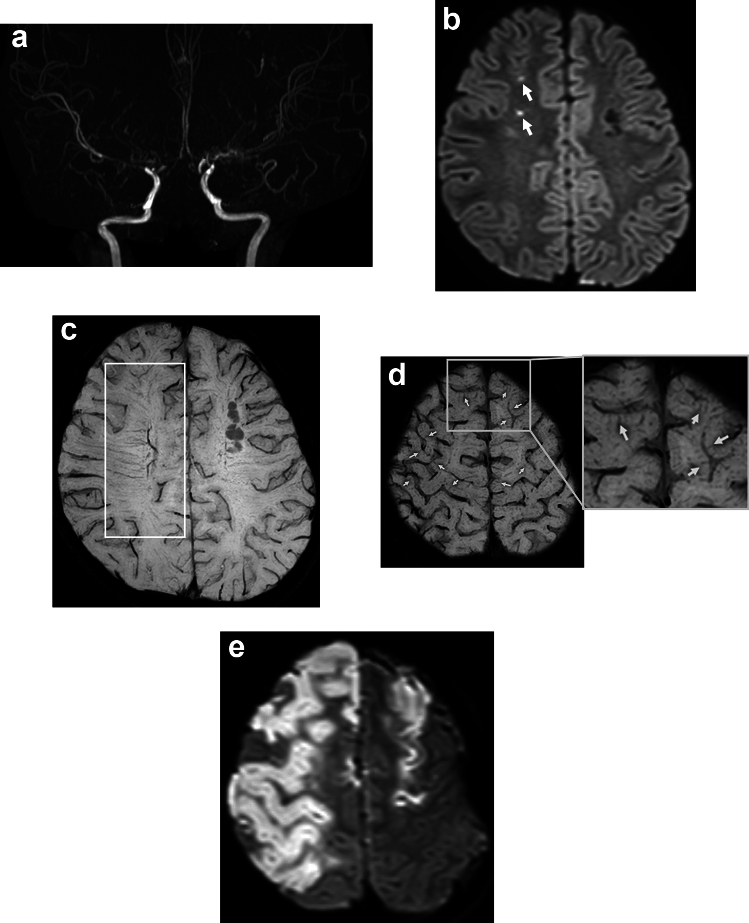
A 7-year-old boy with moyamoya disease presented with atonic seizure. MR angiography shows bilateral internal carotid artery occlusion and moyamoya vessels (**a).** Diffusion-weighted imaging shows small hyperintensities of recent infarct (**b**, arrows). SWI shows the brush sign on the right side (**c**, square). SWI shows multiple linear and fine hypointensities in the bilateral cortex (**d**, arrows), suggesting cortical parenchymal veins. The patient underwent left-sided bypass surgery. Postoperative diffusion-weighted imaging shows large acute infarcts on both sides (**e**)

In MMD, patients with TIA or infarct present with prominent DM, i.e., BS (Fig. 4). It is reported that BS scores strongly correlated with cerebral vascular reserve [3]. This supports the view that BS in MMD essentially indicates increased oxygen extraction as a mechanism, as has been reported in acute cerebral infarct due to large artery occlusion (Fig. 3). Some investigators studied the relationship of BS on preoperative SWI and postoperative infarct [32]. According to their results, preoperative BS and reduced cerebral blood flow were independent risk factors for postoperative infarct. Furthermore, BS showed a significant correlation with higher modified Rankin Scale at discharge. Therefore, the presence of BS on preoperative SWI may be a predictor of infarct after surgical revascularization for MMD (Fig. 5), and this information may contribute to improving surgical outcomes through appropriate perioperative management based on surgical risk stratification.

## Mechanism and clinical implications of brush sign in cerebral venous thrombosis (CVT)

CVT is the presence of a blood clot in the dural venous sinuses, the cerebral veins, or both [33]. Registry-based and cohort studies have shown that CVT primarily affects people under the age of 55, with two-thirds of cases occurring in women [34]. For the diagnosis of CVT, conventional CT or MRI is often performed first in patients with nonspecific acute symptoms or suspicious signs of CVT. In particular, CT venography and MR venography are the methods of choice to confirm the diagnosis of CVT [33]. Some researchers reported that the sensitivity, specificity, negative predictive value, positive predictive value and accuracy of SWI in identifying cortical vein thrombosis were 0.93, 1.0, 1.0, 0.96 and 0.97, respectively, whereas the sensitivity and accuracy of all other sequences were 0.06–0.39 and 0.60–0.73, respectively [35]. Thus, SWI showed the highest sensitivity and accuracy among standard MR sequences including MR venography in detecting early-stage cortical vein thrombosis. However, in cases of subarachnoid hemorrhage or cerebral hematoma, SWI may not be able to identify cortical vein thrombosis. Further studies are needed to verify the performance of SWI in identifying cortical vein thrombosis.

**Fig. 6:**
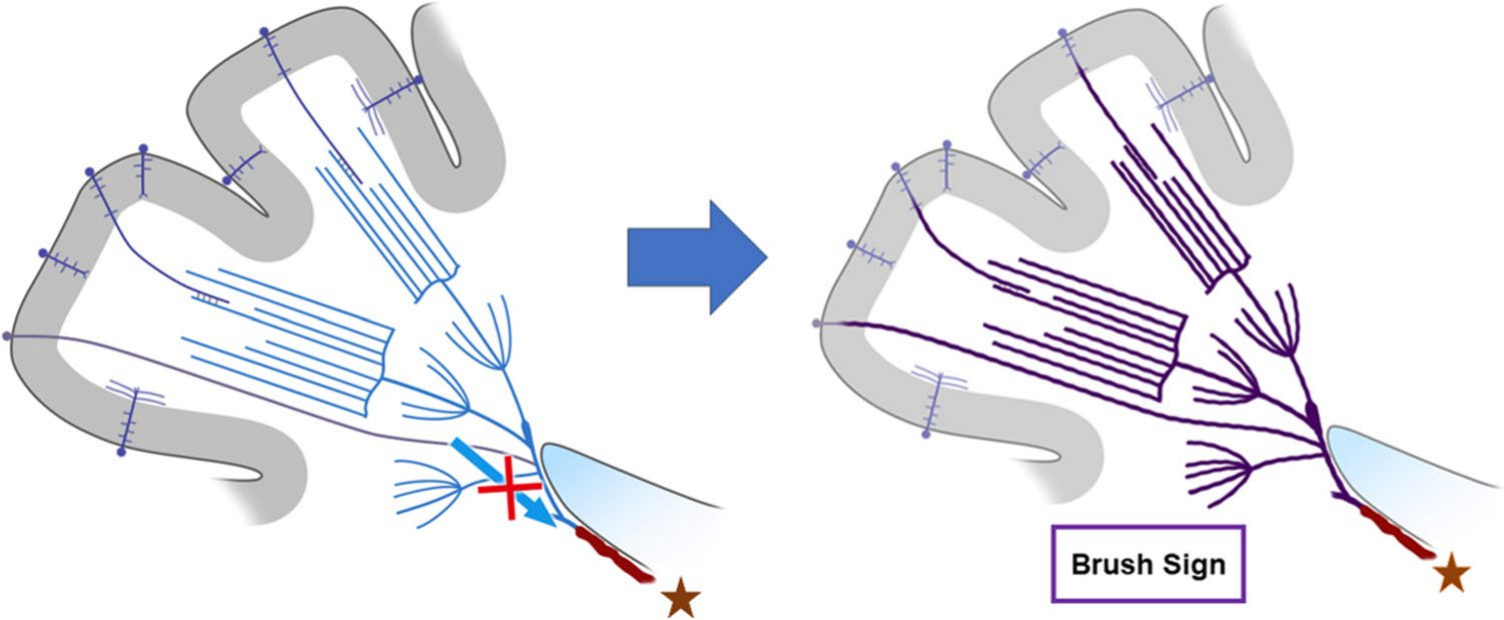
This schematic diagram shows the mechanism of SWI brush sign in cerebral venous thrombosis of the deep venous system (asterisk), which occurs due to congestion and blood oxygenation level-dependent effect of the affected deep medullary and subependymal veins (highlighted in purple)

**Fig. 7:**
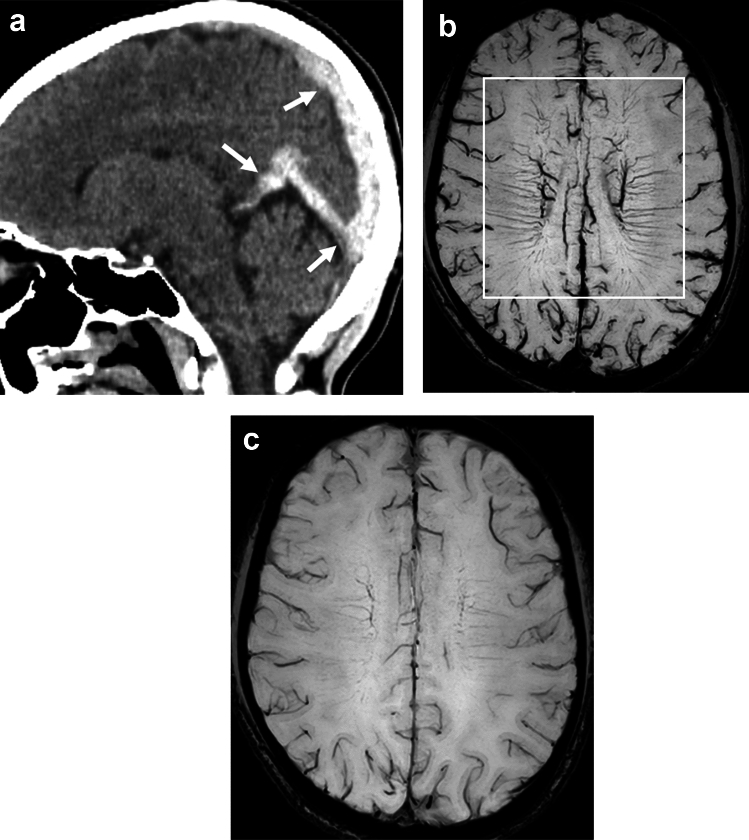
A 21-year-old woman presented with seizure. Two days after onset, CT reveals thrombi in the Galenic, straight, and superior sagittal sinuses (**a**, arrows). SWI shows brush sign (**b**, square) and prominent cortical veins bilaterally. SWI shows disappearance of the brush sign and prominent cortical veins 10 days after anticoagulation treatment (**c**)

The mechanism of BS in CVT of the deep venous system is due to congestion of DM with the formation of collateral circulation between the superficial and deep venous systems of the brain (Fig. 6) and the BOLD effect caused by increased deoxyhemoglobin [4]. BS is fairly frequent in thrombosis of the straight sinus or deep venous system and may, therefore, be useful in identifying this disease. BS is significantly associated with ipsilateral parenchymal brain lesions including edema, degree of thrombus, and development of focal neurological deficits [4]. This suggests that this sign may be a marker of the severity of CVT. It has been reported that BS is frequently observed bilaterally in the CVT cohort and is associated with overt thrombosis of midline venous structures [4] (Fig. 7).

In patients with CVT, venous stasis can increase intracranial pressure, leading to papilledema and permanent vision loss. In one study, the cerebral hemisphere was divided into five regions according to the venous drainage area (superior sagittal sinus, Sylvian vein, transverse sinus and Labé vein, deep cerebral vein, and medullary vein), and if venous prominence was confirmed in one region on SWI, 1 point was added and the results were compared with those of SWI in healthy adults of the same age. It has been reported that the venous stasis score was significantly higher in the hemisphere with papilledema than in the hemisphere without papilledema [36]. Therefore, SWI venous stasis score may be an imaging surrogate marker of elevated intracranial pressure in patients with CVT.

## Mechanism and clinical implications of brush sign in Sturge–Weber syndrome (SWS)

SWS is a neurocutaneous disorder that affects the formation and function of blood vessels in the skin, eyes, and brain. SWS is usually associated with facial capillary malformations in the upper face and leptomeningeal vascular malformations in the brain, which can be seen on contrast-enhanced MRI [37, 38]. Abnormal development of CV is a typical feature, with progressive venous obstruction and associated venous stasis occurring as the disease progresses.

**Fig. 8:**
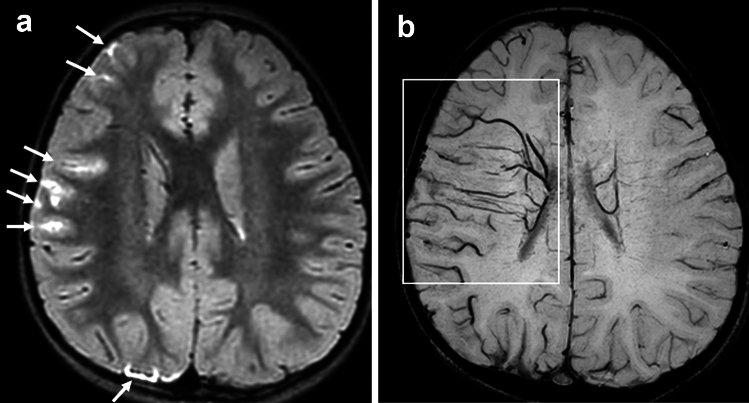
A 3-year-old girl with Sturge–Weber syndrome. She had 5 seizures at age 1 year, but has been seizure-free since taking carbamazepine. Contrast-enhanced FLAIR shows strong enhancement of several sulci on the right side (**a**, arrows). SWI shows enlargement of the deep medullary and subependymal veins on the right side (**b**, square)

**Fig. 9:**
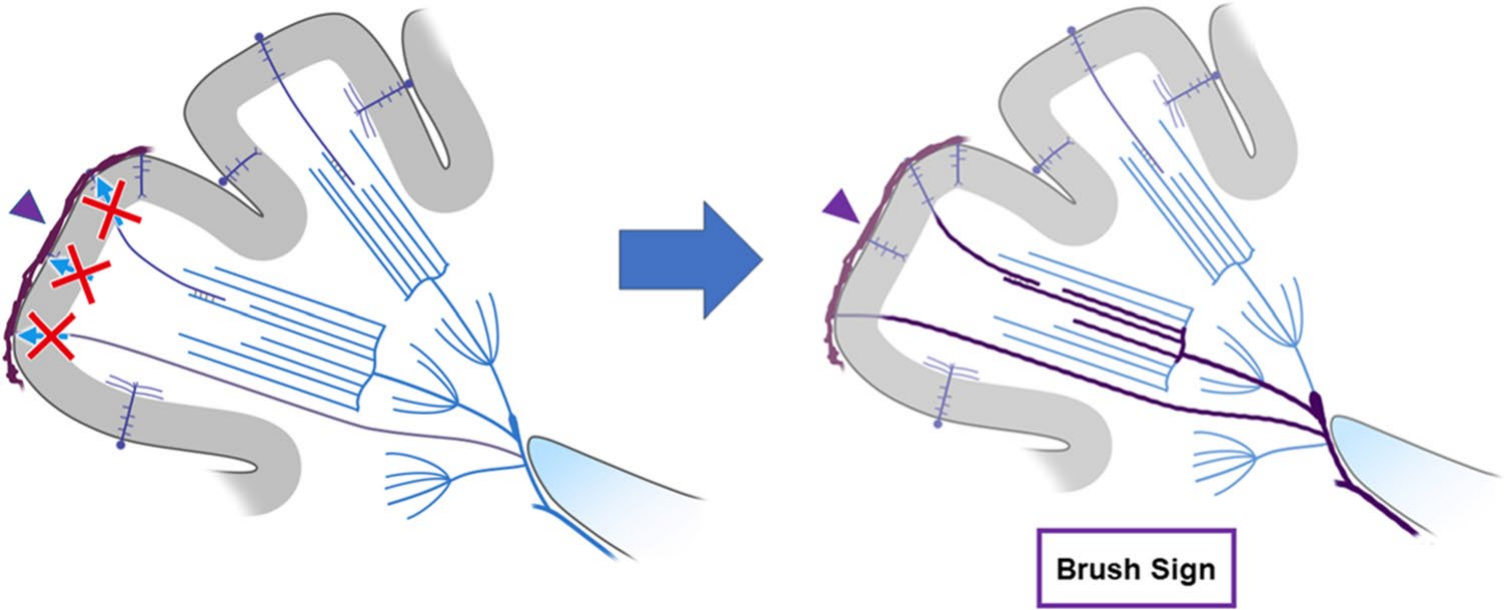
This schematic diagram illustrates the mechanism of the brush sign in Sturge–Weber syndrome. Pial venous malformations (arrowhead) impair superficial venous drainage (x), forming superficial-to-deep collateral drainage pathways and causing engorgement of the deep medullary, transcerebral, and subependymal veins (highlighted in purple)

In a child patient with SWS, collateral veins develop from the superficial veins to the deep venous system, and as the superficial veins are gradually occluded, collateral veins such as enlarged deep medullary veins (EDMV) become increasingly prominent [39] (Fig. 8), which explains the mechanism of BS in SWS (Fig. 9). EDMV have been reported to be present in approximately 73% of SWS patients with epileptic seizures [40, 41], but patients with extensive EDMV have been reported to have fewer epileptic seizures [42]. It has been shown that a child SWS patient with extensive EDMV had good seizure control and better clinical outcomes, despite the presence of severe venous malformations on the brain surface [43]. Thus, EDMV may be a collateral pathway that develops early in the disease and may be partially effective in preventing damage to the adjacent cortex in young patients with SWS.

## Brush sign demonstrated in the cerebral cortex (cortical BS)

**Fig. 10:**
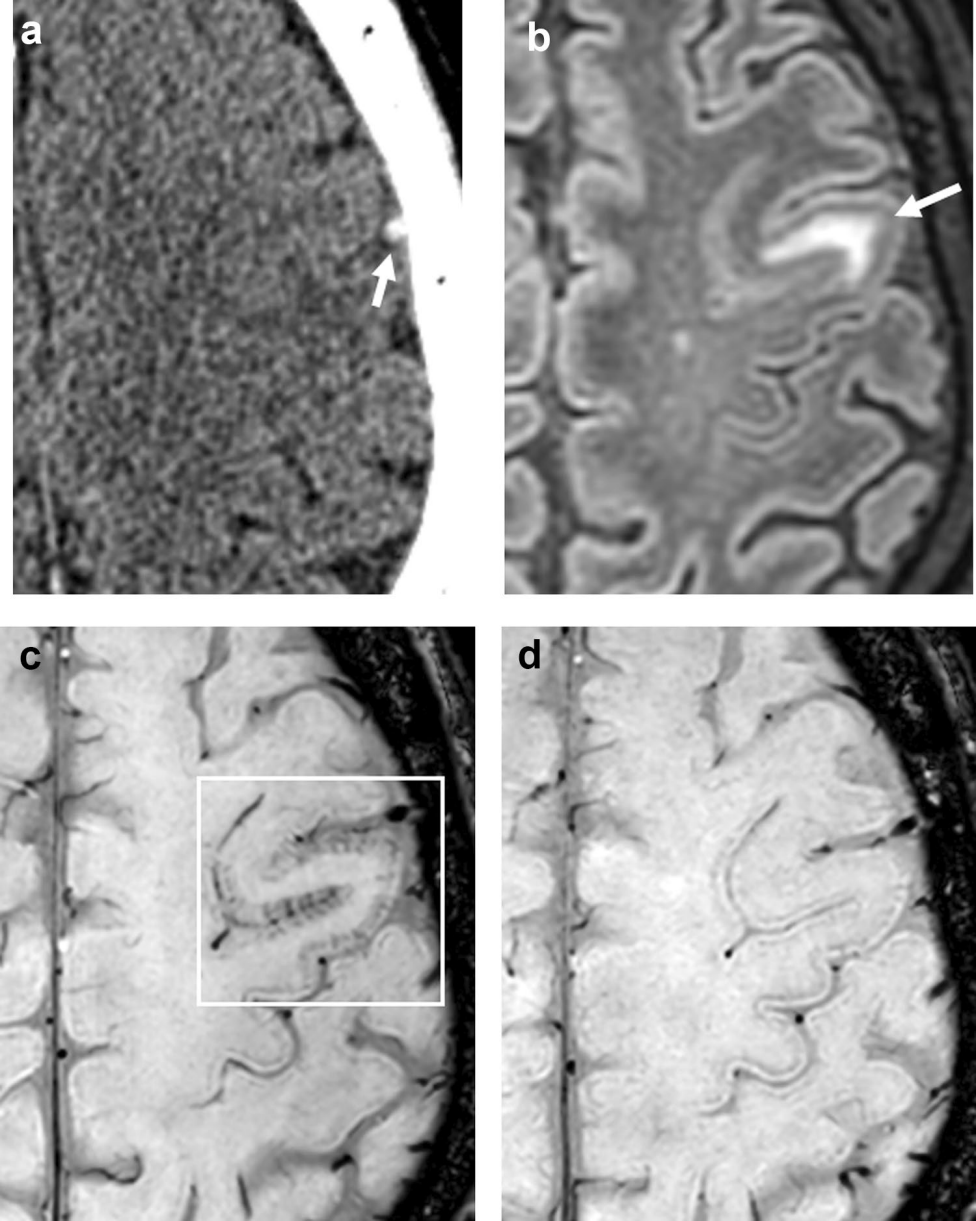
A 60-year-old man presented with headache and seizure. CT and MRI were obtained within 90 min. CT shows a high density of the left cortical vein (**a**, arrow), indicating cortical vein thrombosis. Contrast-enhanced CT revealed thrombus in superior sagittal sinus but patency of the deep venous system (not shown). FLAIR shows edema in the left gyrus (**b**, arrow). SWI shows the cortical brush sign in the affected gyrus (**c**, square). After anticoagulation treatment, SWI shows disappearance of the cortical brush sign (**d**)

**Fig. 11:**
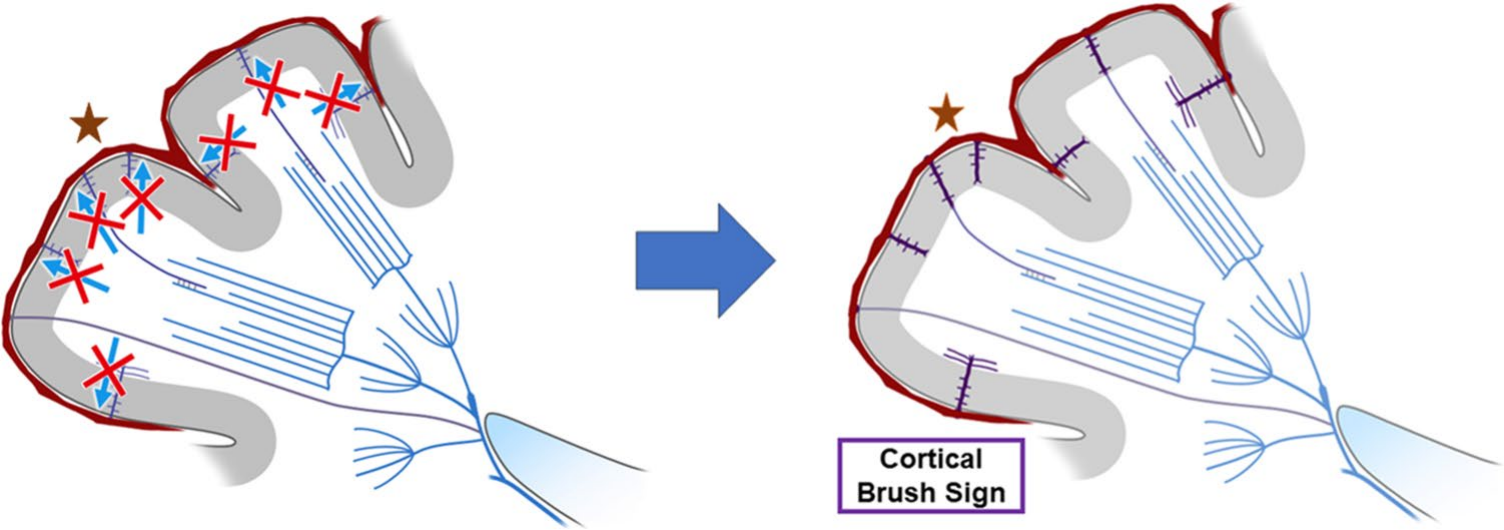
This schematic diagram illustrates the mechanism of the cortical brush sign in cortical vein thrombosis (asterisk). Superficial venous return of the affected cortex is impaired (x), causing these cortical parenchymal veins to become engorged (highlighted in purple). In the absence of deep venous system thrombosis, the deep medullary veins are less engorged

Cortical parenchymal veins, such as intracortical veins, subcortical veins, superficial medullary veins, are very thin and, therefore, usually not visualized on 3 T SWI in healthy subjects, but can be visualized on 7 T SWI [44]. In some stroke patients caused by MMD and CVT, high-resolution SWI at 3 T can also show BS in the cerebral cortex (Figs. 5, 10), and this finding is called cortical BS [45]. The mechanism of cortical BS in MMD can be explained by the BOLD effect of cortical parenchymal veins (Fig. 3), while the mechanism of cortical BS in CVT can be explained by congestion and BOLD effect of cortical parenchymal veins caused by superficial venous thrombosis (Fig. 11).

## Conclusions

The SWI BS has been reported in several CNS diseases, including ischemic stroke, MMD with ischemic events, acute CVT, and SWS. This finding should be kept in mind as it may have clinical implications for each disease.
